# *Ha83*, a Chitin Binding Domain Encoding Gene, Is Important to *Helicoverpa armigera* Nucleopolyhedrovirus Budded Virus Production and Occlusion Body Assembling

**DOI:** 10.1038/srep11088

**Published:** 2015-06-09

**Authors:** Huan Yu, Jian Xu, Qiang Liu, Tong-Xian Liu, Dun Wang

**Affiliations:** 1 State Key Laboratory of Crop Stress Biology for Arid Areas Northwest A&F University, Yangling, Shaanxi, P. R. China; 2 Key Laboratory of Applied Entomology, Northwest A&F University, Yangling, Shaanxi, P. R. China

## Abstract

*Helicoerpa armigera* nucleopolyhedrovirus (HearNPV) ha83 is a late expressed gene that encodes a chitin binding protein. Chitin domain truncation studies revealed that the cysteine at the 128 amino acid position probably played an important role in both chitin binding ability and protein transmission of Ha83. In order to study the function of *ha83* in the HearNPV infection cycle, an *ha83* knockout HearNPV (Ha83KO) was constructed via homologous recombination. Viral growth and viral DNA replication curves showed that fewer budded virions were produced in Ha83KO transfected cells, while viral DNA replication was increased. Electron microscopy revealed that fewer nucleocapsids were transmitted from virogenic stroma in the Ha83KO transfected cell nucleus, and the morphology of occlusion bodies was prominently larger and cube-shaped. Furthermore, DNA quantity in occlusion bodies of Ha83KO was significantly lower than the occlusion bodies of HaWT. The transcription analysis indicated that these changes may be due to the decreased expression level of viral structural associated genes, such as *polyhedrin*, *p10*, *pif-2*, or *cg30* in Ha83KO infected cells. Above results demonstrated that the cysteine at the 128 amino acid position in Ha83 might be the key amino acid, and Ha83 plays an important role in BVs production and OBs assembling.

The *Baculoviridae* encompasses a diverse group of insect specific DNA viruses that have been reported worldwide, predominantly from insects of the orders Lepidoptera, Hymenoptera, and Diptera^1^. Two virion phenotypes, budded viruses (BVs) and occlusion-derived viruses (ODVs), are produced during the biphasic infection process of baculoviruses. Although BVs and ODVs have a common nucleocapsid structure and carry identical genetic information, they differ in the composition of their envelopes^2^,^3^,^4^. In the insect hosts, BVs induce the viral infection between the tissues and ODVs transmit the virus among the host individuals. ODVs form in the peristromal space or intranuclear ring zone of the baculovirus-infected cell nucleus and occluded in occlusion bodies (OBs)^5^.

*Helicoverpa armigera* nucleopolyhedrovirus (HearNPV) is a species type of *Alphabaculovirus* in the *Baculoviridae* and has been completely sequenced^6^. HearNPV presents a genome of 131 kbp, which contains 135 predicted open reading frames (ORFs)^6^. Several HearNPV genes, such as the ecdysteroid UDP-glucosyltransferase gene (*egt*)^7^, the late expression factor 2 gene (*lef-2*)^8^, *chitinase*^9^, *ha81*^10^, *p10*^11^, and the F- protein gene (*ha133*)^12^ have been characterized. HearNPV orf83 (*ha83*) as a chitin binding domain encoded gene in HearNPV has no homologue that shares over 30% identity in nucleotide sequence when comparing with the genomic sequenced NPVs and GVs except for HezeNPV and SpexNPV. Ha83 protein is a nonstructural protein revealed to contain a conserved chitin binding domain (CBD) in the C-terminal. The protein is first detected in the cytoplasm of infected HzAM1 cells at 12 h post infection (h p.i.) then trafficked into the nucleus at 48 h p.i.^13^. *Ha83* is predicted to code for 18.8 kDa protein but its function in the viral life cycle remains unknown.

To determine the role of *ha83* in HearNPV replication and BVs production, a HearNPV bacmid was used to generate an *ha83* deletion mutant. We found that *ha83* is involved in BV production for the deletion mutant and produces fewer BVs than the wild type in one step viral growth curve analysis. However, viral DNA replication was increased by the deletion of *ha83* in the quantitative PCR analysis. Electromicroscopy observation showed that *ha83* is not required for nucleocapsid formation but it may affect polyhedra morphology. To investigate the functional role of CBD in the Ha83 subcellular transport and chitin binding activity, eukaryotic expression vectors and prokaryotic expression vectors were constructed to truncate the C-terminus region, a cysteine-rich region. The transient eukaryotic expression vectors contained the truncated *ha83* fused with *egfp* and were transfected into HzAM1 cells and confocal microscopic analysis showed that, in late infection, the first N-terminal 141 amino acids were necessary for the traffic of Ha83 from the cytoplasm into the nucleus. The truncated prokaryotic purified proteins also revealed a similar result that the conserved cysteine at the 128 amino acid position was important to Ha83 chitin binding activity.

## Results

### Transcriptional mapping of the 3′ and 5′ end of *ha83* transcripts

**Figure 1:**
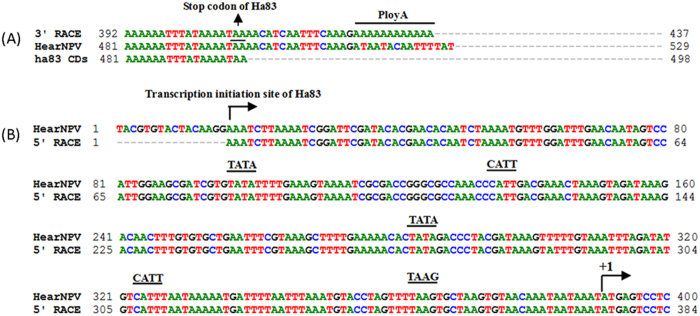
3′RACE and 5′RACE analysis of *ha83* in HearNPV infected HzAM1 cells. (**A**) 3′RACE analysis of *ha83* in HearNPV infected HzAM1 cells at 48 h p.i. The arrow points to the location of TAA stop codon. The poly A tail addition site is underlined. (**B**) Sequences located at the 5′end of *ha83*. The TATA box, ATAAG, TAGG, and CATT promoter motifs are underlined. The rightward arrow indicates the location of the transcription initiation sites. The arrow points to the ATG translation start codon of *ha83*.

The 3′ Rapid Amplification of cDNA Ends (RACE) obtained sequences mapped the 3′end of the *ha83* transcript at 16 nucleotides (nt) downstream of the putative translation stop codon TAA and 13 nt downstream of the last A of the polyadenylation signal sequence AATAAA, which is overlapped with the stop codon TAA (Fig. 1A). Ten clones were sequenced from 5′ RACE and the *ha83* mRNA putative start sites were identified at 374 nt upstream of the ATG codon at three contiguous A residues (Fig. 1B). A search of the 374 nt 5′ untranslated regions (UTR) showed that there are two TATA boxes located at positions −107 and −293 upstream of the ATG; a late strong promoter ATAAG element was at position −202; another TAAG late promoter element was found at −27; five early promoter CATT elements were found at −67, −187, −211, −255, and −310 upstream of the ATG (Fig. 1B).

### Expression of truncated His-Ha83s and chitin binding assays

**Figure 2:**
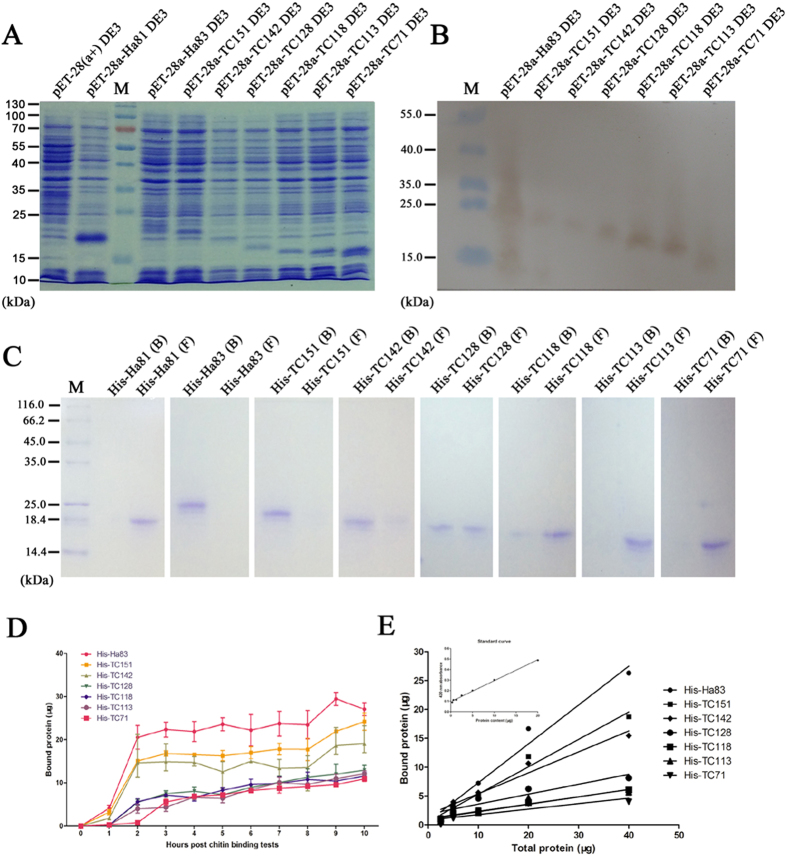
Chitin binding tests on Ha83 and truncated mutants. (**A**) SDS-PAGE investigation of Ha83 and truncated mutants expressed by *E. coli* BL21(DE3). The protein samples were separated by 15% SDS-PAGE. (**B**) Western blot analyses of Ha83 and truncated mutants expressed by *E. coli* BL21(DE3). The protein samples were first separated by 10% Tricine-SDS-PAGE and then transferred to a nitrocellulose membrane. A monoclonal anti-His tag antibody (1:4000) (Abcam, GBR) was used as the primary antibodies and the signal was visualized with an Enhanced HRP-DAB Chromogenic Substrate Kit (TIANGEN, CHN). (**C**) Chitin binding tests of Ha83 and truncated mutants incubated with chitin colloid for 16 h at 4 ℃. The bound protein (**B**) and unbound protein in the supernatant (**F**) was first separated by centrifugation then lyophilized and loaded in each lane of a 10% Tricine-SDS-PAGE system. The gels were stained with Coomassie Blue (Sigma). In order to improve the clarity and conciseness, the figures exhibited here were cropped; the full-length gels or blots are presented in Supplementary Figure S2–5. (**D**) Temporal course of protein samples binding to crab shell chitin. Each chitin binding test was performed in triplicate and average quantity of bound protein was derived from three data measurements for each time point post incubation. Error bars represent the standard deviation. (**E**) Double-reciprocal plot of protein binding to chitin. A standard curve of Ha83 quantity and absorbance figure is zoomed out in the left up corner. The slope and R^2^ value of each reciprocal line of protein samples are shown in the table at the bottom of the figure.

To identify the functional cysteine needed for the chitin binding domain in Ha83, we constructed seven pET-28a(+) plasmids containing different parts of HearNPV Ha83 fused with His-tag, as well as an *ha81* expression vector to express Ha81 used as a negative control. Clear bands with molecular weights about 22.2 kDa (His-Ha83), 20.6 kDa (His-TC151), 19.6 kDa (His-TC142), 18.1 kDa (His-TC128), 17.0 kDa (His-TC118), 16.4 kDa (His-TC113), 15.0 kDa (His-TC71), and 17.6 kDa (His-Ha81) were separated in PAGE gel stained with coomassie blue (Fig. 2A). The His-tag antibody detected specific bands in close agreement with those of target bands in the coomassie blue stained PAGE gel (Fig. 2B).

The purified His-tag fused proteins were incubated with chitin colloid for 16 h to perform the chitin binding assay. Distinct bound protein bands were found from His-Ha83 and His-TC71 tested lanes in the 10% tricine SDS-PAGE gel (Fig. 2C). For His-TC128, a clear band emerged in the unbound protein loaded lane, as well as in the bounded protein loaded lane (Fig. 2C). The following mutants that lost two or more conserved cysteines showed a weak ability to bind with chitin (Fig. 2C): The Ha81 did not bind with the chitin colloid as no band was found at the bound lane, but a strong band was found at the unbound lane (Fig. 2C). These results demonstrated that conserved cysteines were related to the Ha83 chitin binding activity.

To further investigate the chitin binding capacity and ability of different mutants, each of the proteins (His-Ha83, His-TC151, His- TC142, His- TC128, His- TC118, His- TC113, and His- TC71) were mixed with a certain quantity of chitin colloid. The chitin binding capacity was first determined by the incubation time (Fig. 2D). After ten hours, the highest binding capacity was ascertained for His-Ha83 (0.044 μM or 0.97 μg proteins/mg chitin) followed by His-TC151 (0.038 μM or 0.81 μg proteins/mg chitin), His-TC142 (0.032 μM or 0.63 μg proteins/mg chitin), His-TC128 (0.024 μM or 0.43 μg proteins/mg chitin), His-TC118 (0.023 μM or 0.38 μg proteins/mg chitin), His-TC113 (0.023 μM or 0.37 μg proteins/mg chitin), and His-TC71 (0.024 μM or 0.36 μg proteins/mg chitin). The chitin binding ability was then determined by equilibration (16 h) of different quantities of tested protein with a chitin colloid sample. The dissociation constants (K_d_) were determined by a double-reciprocal plot (Fig. 2E). The slope of the regression lines decreased with the cysteines truncation (Fig. 2E). The highest K_d_ value was obtained for His-TC71 (K_d_ of 7.6 μM) and the lowest for His-Ha83 (K_d_ of 0.62 μM), which implied that His-Ha83 binds about 13 times more effectively than His-TC71.

### Residue cysteine 128 is important for *ha83* traffic from the cytoplasm to the nucleus

**Figure 3:**
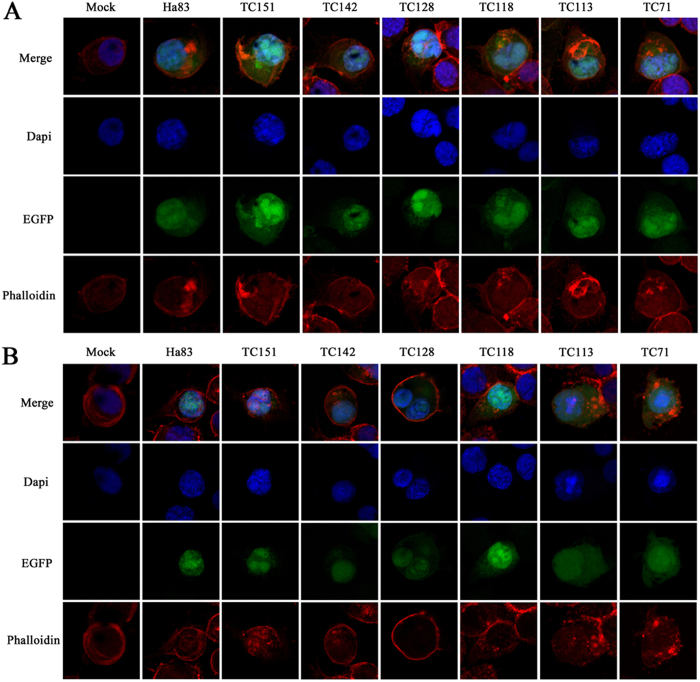
Subcellular localization of Ha83 and truncated mutants as demonstrated by confocal microscopy. HzAM1 cells were co-transfected with HaBacHZ8 bacmid DNA and pGE-Ha83, pGE-TC151, pGE-TC142, pGE-TC128, pGE-TC118, pGE-TC113, and pGE-TC71, respectively. At the designated time points, cells were fixed, inmmunostained with TRITC-labeled phalloidine to stain the cytoskeleton protein (red) and 4′,6-diamidino-2-phenylindole (Dapi) to stain the nucleus (blue). Mock cells were used as a control. (**A**) Subcellular localization of Ha83 and truncated mutants at 24 h p.t. (**B**) Subcellular localization of Ha83 and truncated mutants at 48 h p.t.

To further characterize the function of the Ha83 chitin binding domain in the baculovirus life cycle, the subcellular localization of Ha83 and truncated mutants were analyzed by confocal fluorescence microscopy. All transfected cells showed only background level fluorescence at 6 h p.t. and were observed to contain green fluorescence in the cytoplasm at 12 h p.t. (data not shown). At 24 h p.t., all transfected cells contained green fluorescence evenly in the nucleus and cytoplasm, which indicated that Ha83 began to traffic from the cytoplasm into the nucleus at this point of time without distinction of truncation (Fig. 3A). However, at 48 h p.t., the EGFP fused protein in pGE-Ha83/HaBacHZ8 co-transfected cells were all detected in the nucleus, which indicated that all Ha83 were located in the nucleus (Fig. 3B). Following that, in pGE-TC151 and pGE-TC142 co-transfected cells, target proteins were predominantly localized within the nucleus, with levels of green fluorescence detected in the cytoplasm (Fig. 3B). However, pGE-TC128, pGE-TC118, pGE-TC113, and pGE-TC71 co-transfected HzMA1 cells showed dispersal EGFP fluorescence at the same point of time (Fig. 3B). Whereas, Ha81, which was selected as a negative control, was reported to be located in the cytoplasm from 24 to 48 h.p.t. (10). These results suggested that the cysteine at the 128 amino acid position probably play an important role in the Ha83 protein traffic from cytoplasm to nucleus.

### Deletion of *ha83* affects BV production

**Figure 4:**
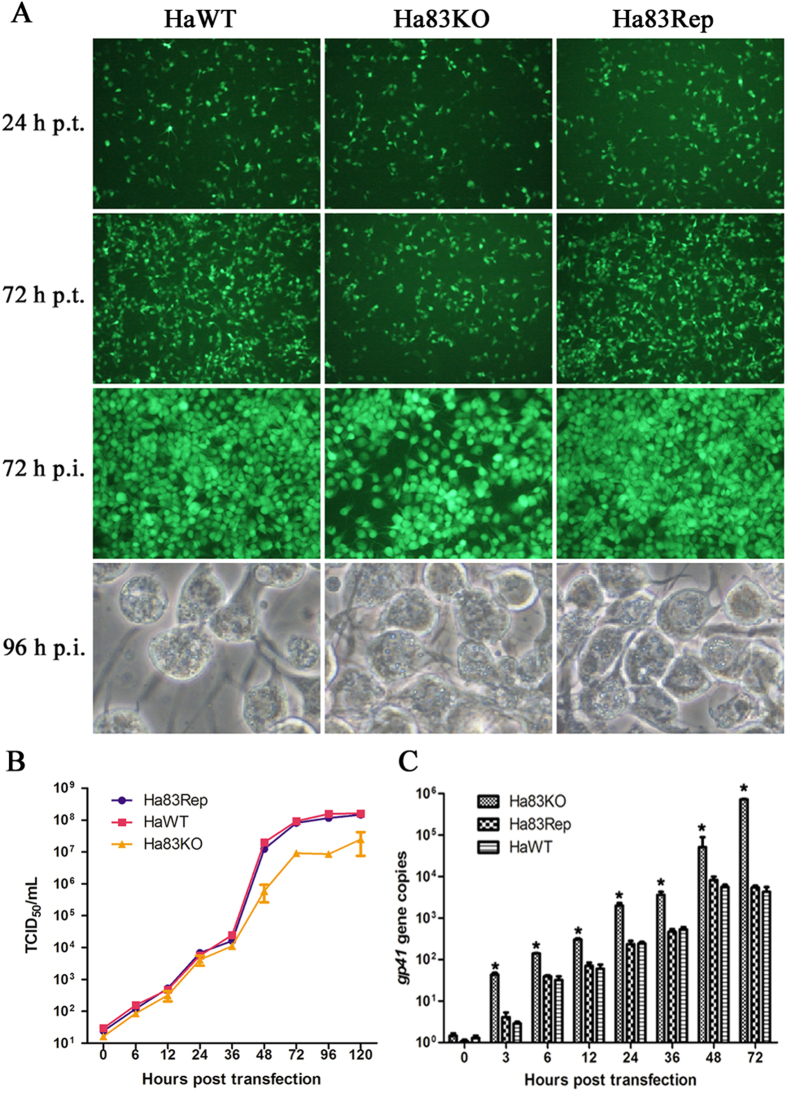
*Ha83* deletion analysis in HzAM1 cells. (**A**) Microscopy analysis of viral replication in HzAM1 cells. Fluorescence microscopy shows the progression of viral infection in HzAM1 cells transfected with HaWT (*polyhedrin* repaired HaBacHZ8 bacmid), Ha83KO (*ha83* deleted HearNPV mutant), and Ha83Rep (Ha83KO reinserted a *ha83* in the *polyhedrin* locus) from 24 to 72 h p.t. An additional infection fluorescence microscopy shows the infectivity of viral infection in HzAM1 cells infected with HaWT, Ha83KO, and Ha83Rep at 72 h p.i. Light microscopy shows the formation of occlusion bodies in HaWT, Ha83KO, and Ha83Rep infected cells at 96 h p.i. (**B**) Virus growth curves as determined by TCID_50_ endpoint dilution assays. For the transfection growth curves, HzAM1 cells were transfected with HaWT, Ha83KO, and Ha83Rep bacmid DNA. The supernatants were then harvested at the indicated time points p. t., and titers were determined using TCID_50_ assays. Each data point was determined from the average of three independent transfections, and error bars represent the standard deviations. (**C**) Real-time PCR analysis of viral DNA replication. HzAM1 cells were transfected with each bacmid DNA (HaWT, Ha83KO, or Ha83Rep). At the designated time points, total intracellular DNA was extracted and analyzed by real-time PCR. The y-axis value indicates the number of viral DNA genome copies within each sample. The graph shows the results of three independent replication assays and each replication was tested for another three times, with error bars indicating the standard deviations. Statistically significant differences are indicated by asterisks (*P ⊠ 0.05).

DNA from Ha83KO (the *ha83* knockout HearNPV mutant), Ha83Rep (Ha83KO with *ha83* repaired at the *polyhedrin* locus), and HaWT (a positive control bacmid constructed by *polyhedrin* reintroduction into HaBacHZ8) were individually transfected into HzAM1 cells, and cells were observed for cytopathic effects and EGFP expression. At 24 h p.t., 15 to 25% of Ha83KO, Ha83Rep, and HaWT DNA transfected cells were expressing EGFP, corresponding to cells initially transfected with bacmid DNA (Fig. 4A). At 72 h p.t., 40 to 60% of Ha83Rep and HaWT DNA transfected cells expressed EGFP, whereas in Ha83KO DNA transfected cells, only 30 to 50% of cells expressed EGFP (Fig. 4A). The increase in fluorescent cell number suggested that Ha83KO, Ha83Rep, and HaWT DNA transfected cells can produce infectious BV, which led to the infection of untransfected cells. On the other hand, Ha83KO DNA transfected cells appeared to produce fewer infectious BVs to support secondary infections leading to EGFP expressed cells.

To determine infectious budded virus production, supernantant from Ha83KO, Ha83Rep, and HaWT DNA transfected cells were harvested at 96 h p.t. and used to treat uninfected HzAM1 cells. By 72 h p.i., the majority of the Ha83Rep or HaWT infected cells expressed EGFP, whereas in Ha83KO DNA infected cells, only 75% expressed EGFP, which confirmed that *ha83* deletion affected infectious BV production (Fig. 4A).

Microscopy analyses revealed OBs in infected cells when all of the three constructs were used, and no differences in the numbers of cells containing OBs were observed at 48 h p.i. (data not shown). By 96 h p.i., most of the Ha83Rep and HaWT infected cells contained OBs, whereas fewer of the Ha83KO infected cells contained OBs (Fig. 4A). The infection experiments showed that deletion of *ha83* led to a decrease in the production of infectious BVs but did not affect OB formation.

To further assess the effect of *ha83* deletion on BVs production, a one step viral growth curve was performed. As expected, the supernatants from HzAM1 cells transfected with Ha83Rep and HaWT revealed a steady increase in virus production, and the *ha83* repaired virus showed similar growth kinetics in titer to the WT virus (Fig. 4B). Statistical analysis revealed that Ha83KO produced BVs to a significantly lower titer than HaWT (F = 110.56, d.f. = 2, 4; *P* < 0.0001) and the *ha83* rescue virus Ha83Rep (F = 110.56, d.f. = 2, 4; *P* < 0.0001).

### Viral DNA replication in HzAM1 cells

To determine whether *ha83* was essential for viral DNA replication, quantitative PCR analysis was performed to compare the initiation and levels of viral DNA replication in Ha83KO, Ha83Rep, and HaWT transfected cells. Equal amounts of virus-transfected HzAM1 cells were collected at designated time points, and viral DNA was prepared as described above. Results showed that for these three viruses, the levels of DNA replication started to increase from 24 h p.t., and there were no significant differences between Ha83Rep and HaWT DNA transfected cells at any point (Fig. 4C). However, Ha83KO DNA transfected cells showed a significantly higher number of viral DNA copies than the *ha83* rescue virus Ha83Rep (F = 336.12, d.f. = 2, 4; *P* < 0.0001) and HaWT (F = 336.12, d.f. = 2, 4; *P* < 0.0001) at each selected time point.

### *Ha83* is critical for polyhedra formation

To further investigate the lower BV production in the *ha83* knockout virus and to analyze whether the deletion of *ha83* has any effect of virus morphogenesis, electron microscopy analyses were performed on Ha83KO, Ha83Rep, and HaWT transfected HzAM1 cells.

**Figure 5:**
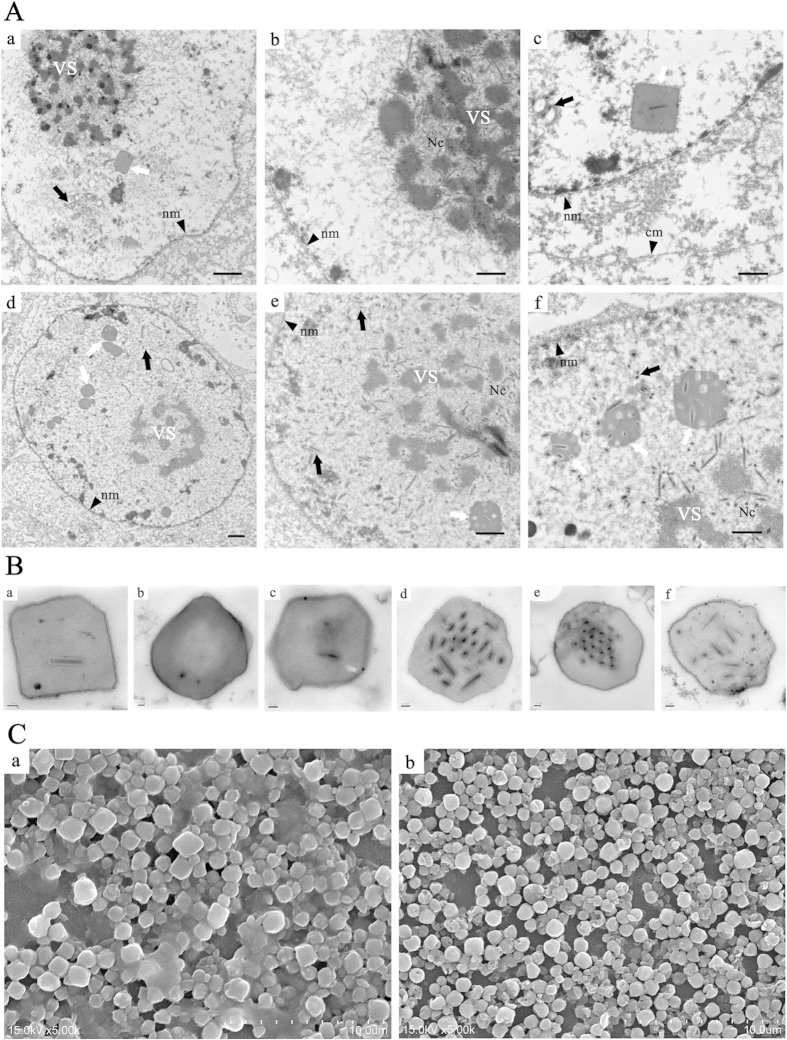
Occlusion bodies assembling and morphology analysis of Ha83KO and HaWT. (**A**) TEM analyses of HzAM1 cells transfected with Ha83KO and HaWT DNA. (**a**) A square shape occlusion body (white arrows) and normal nucleocapsids (black arrows) formed in the nucleus of Ha83KO DNA transfected cells. (**b**) Electron-dense nucleocapsids in the virogenic stroma (VS). (**c**) A square shaped occlusion body with only one ODV inside and few nucleocapsids in the nucleus of Ha83KO DNA transfected cells. (**d**) Occlusion bodies and rod shaped nucleocapsids are formed in the nucleus of HaWT DNA transfected cells. (**e**) Electron-dense nucleocapsids in the VS. (**f**) Normal polyhedron shape occlusion bodies and numerous nucleocapsids in the nucleus of HaWT DNA transfected cells. Nm, nuclear membrane. Cm, cytoplasm membrane. Scale bar, (**a**) and (**c**), 1 μm, others 500 nm. (**B**) TEM analyses of purified occlusion bodies from Ha83KO and HaWT infected larvae. (**a–c**) Occlusion bodies purified from Ha83KO infected larvae are mostly square shaped with several ODVs inside. **(d–f**) Occlusion bodies purified from HaWT infected larvae are normal shaped with dozens of ODVs inside. Scale bar, 100 nm. (**C**) SEM analyses of purified occlusion bodies from Ha83KO or HaWT infected larvae. (**a**) Occlusion bodies purified from Ha83KO infected larvae are cubic in morphology. (**b**) Occlusion bodies purified from HaWT infected larvae are standard polyhedron shape in morphology. Scale bar, 10 μm.

As expected, cells transfected with the control bacmid, Ha83Rep, showed typical characteristics of a HearNPV infection at 72 h p.i., including a net-shaped virogenic stroma (VS) inundated with electron-dense rod-shaped nucleocapsids (Fig. 5Ae,f), numerous nucleocapsids transported from the VS to nuclear membrane (Fig. 5Ae), and polyhedra containing mature virions in the ring zone (Fig. 5Af). Cells transfected with HaWT showed characteristics similar to those transfected with Ha83Rep (data not shown).

In Ha83KO bacmid transfected cells, a well-defined VS and abundant electron-dense rod-shaped nucleocapsids were observed (Fig. 5Ab) and the nucleocapsids were morphologically indistinguishable from those observed in HaWT (data not shown) and Ha83Rep transfected cells (Fig. 5Ae). However, most of the nucleocapsids were observed in the VS, and seldom were transported from the VS to nuclear membrane (Fig. 5Aa,b,c). Consequently, even though the shape and size of the polyhedra were comparable to those in WT bacmid transfected cells (data not shown) and repair bacmid transfected cells (Fig. 5Ad,e,f), the shape of polyhedra observed in the Ha83KO bacmid transfected cells turned into a square and appeared to contain fewer ODVs (Fig. 5Aa,c).

Additional TEM observation of polyhedra purified from Ha83KO and HaWT infected larvae showed similar morphology changes and ODV quantity of OBs as described above (Fig. 5B). The shape of polyhedra was further verified by scanning electron microscopy (Fig. 5C). Ha83KO OBs had diameters between 0.71 and 1.62 μm with an average (±SE) value of 1.11 ± 0.19 μm (N = 80), which was significantly larger than the diameter of HaWT OBs that ranged from 0.64 to 1.24 μm with an average value of 0.89 ± 0.13 μm (N = 80) (t = 24.39, df = 79, *P* < 0.0001). Ha83KO OBs were 25% larger than those of HaWT OBs on average diameter . In addition, the morphological form of Ha83KO OBs had a cubic shape compared to the polyhedral form of wild type OBs (Fig. 5).

### Ha83KO bacmid OBs have a decreased DNA content

DNA content was quantified by qPCR (efficiency = 101.6%, r^2^ = 0.995). The presence of non-specific amplification resulting in the high PCR efficiency was considered unimportant, as only one peak could be observed in the melting curve. Significant differences were observed in the mean concentration of DNA in OB samples from Ha83KO, Ha83Rep and HaWT (F = 5.833, d.f. = 2, 4, *P* = 0.0134). Ha83KO OBs yielded an average (±SE) of 0.18 ± 0.29 ng DNA/10^6^ OBs, significantly less than HaWT (19.35 ± 13.88 ng DNA/10^6^ OBs) and Ha83Rep OBs (17.13 ± 12.10 ng DNA/10^6^ OBs) (Fig. 5B). For HearNPV, a single nucleocapsid nucleopolyhedrovirus, the DNA copy number presented the ODV number contained in OBs. In this case, the ODV contained in Ha83KO OBs (56.7 ± 91.4 copies/OB) was approximately a hundred fold less than HaWT OBs (6096.1 ± 4372.8 copies/OB) and Ha83Rep OBs (5396.7 ± 3812.0 copies/OB).

### Quantification of transcripts from selected viral genes by qPCR

**Figure 6:**
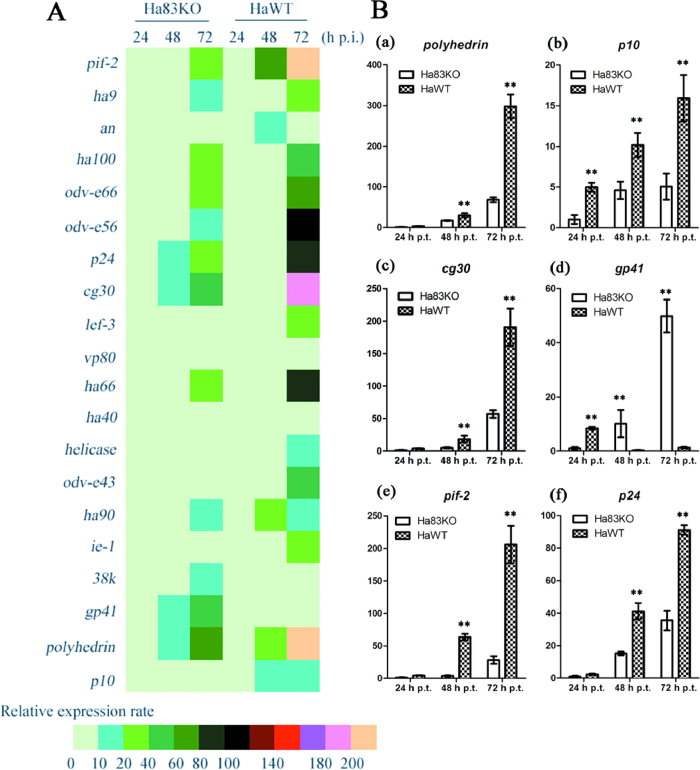
Relative expression of viral genes in Ha83KO and HaWT transfected HzAM1 cells. (**A**) Heatmaps of selected gene expression in Ha83KO or HaWT transfected HzAM1 cells at 24, 48, and 72 h p.t. (**B**) Quantification of transcripts from selected viral genes by qPCR. (**a**) *polyhedrin*, (**b**) *p10*, (**c**) *cg30*, (**d**) *gp41*, (**e**) *pif-2*, (**f**) *p24*. For each graph, the y-axis indicates the relative amount of RNA estimated in comparison to 1.0 μg of RNA purified from infected HzAM1 cells. Error bars represent the standard deviation. Statistically significant differences between the *ha83* knockout and control virus are indicated by asterisks (**P < 0.01).

To analyze the effects of the *ha83* knockout, we examined the mRNA accumulation of several virus structural protein associated genes. For the target genes, we selected *polyhedrin* and *p10* as representative occlusion body associated structural genes, and *pif-2*, *ha9*, *an*, *ha100*, *odv-e66*, *odv-e56*, *p24*, *cg30*, *lef-3*, *vp80*, *ha66*, *ha40*, *helicase*, *odv-e43*, *ha90*, *ie-1*, *38k*, *gp41* as representative ODV or BV associated structural genes. Quantitative PCR analyses showed that, with the exception of *an*, *vp80*, *ha40* and *38k*, the relative expression levels of selected genes changed dramatically in Ha83KO virus transfected cells compared to HaWT virus transfected cells (Fig. 6A). The expression of *polyhedrin* and *p10* in Ha83KO transfected cells were significantly lower than HaWT transfected cells, with a 77% reduction in *polyhedrin* expression (Fig. 6Ba) (F = 607.94, d.f. = 1, 48; *P* < 0.0001) and 68% reduction in *p10* expression (Fig. 6Bb) (F = 235.45, d.f. = 1, 48; *P* < 0.0001). This indicated that the deletion affected the polyhedra associated genes expression levels. In the case of *p24* and *cg30*, for example, most of the ODV or BV structural associated gene expression were blocked by the deletion of *ha83* (Fig. 6A). *p24* was significantly affected by the deletion (F = 859.45, d.f. = 1, 48; *P* < 0.0001) and showed a 61% reduction at 72 h p.t. (Fig. 8Bf). *cg30* showed a 70% reduction at 72 h p.t. (Fig. 6Bc) and was also significantly blocked in gene expression level (F = 226.32, d.f. = 1, 48; *P* < 0.0001). The expression levels of *pif-2* in the Ha83KO transfected cells at 72 h p.t. showed the most dramatic (86%) reduction (Fig. 6Be) (F = 586.05, d.f. = 1, 48; *P* < 0.0001). However, *gp41* in Ha83KO transfected cells pronounced a significantly higher expression level than detected in HaWT transfected cells (Fig. 6Bd) (F = 373.70, d.f. = 1, 48; *P* < 0.0001). *gp41* mean (±SE) relative expression was 49.87 ± 6.02 for cells transfected with Ha83KO, whereas 1.29 ± 0.29 was recorded for cells transfected with HaWT, indicating that *gp41* expressed approximately 39 fold higher after *ha83* deletion. The entire selected genes expression analysis can be found as Supplementary Figure S1 online.

## Discussion

HearNPV *ha83* has been identified as one of the 20 unique genes^6^, which share no more than 30% amino acid sequence identity with other baculovirus^13^. Our studies have shown that *ha83* is important for BV production and the assembling of normal OBs; and that the cysteine at 128 amino acid position in Ha83 chitin binding domain plays an important role in chitin binding activity and protein transmission.

5′ RACE analysis showed that the transcription initiation site of *ha83* is at 374 nt upstream of ATG, and this long promoter contains many well characterized promoter elements. Although *ha83* has been reported as a late expression gene, there are five CATT early promoter elements within the 5′ UTR of *ha83*^13^, whereas a similar inconsistent early gene *ac78* with two consensus baculovirus late gene promoter motif TAAG has been reported in AucaMNPV^14^. The products of baculovirus early genes are often involved in DNA replication, regulation of late gene expression and host-modification processes^15^.

Ha83 contains a predicted type II chitin binding domain at the C terminal which is characterized by the presence of a 6-cysteine motif with the consensus sequence C-X_13–20_-C-X_5–6_-C-X_9–19_-C-X_10–14_-C-X_4–14_-C (where X is any amino acid other than cysteine)^16^. Detailed analysis of the six truncated mutants of Ha83 suggested that the cysteine at the 128 aa position might be the key amino acid in the Ha83 CBD, for the truncated mutant without the cysteine128 showed weak ability to bind with chitin colloid or protein subcellular traffic (Fig. 2, Fig. 3). The type II CBD is referred to as the peritrophin-A domain because it is found in peritrophin-A chitin-binding proteins, particularly in the peritrophins of insect peritrophic membranes (PM)^16^, such as Ac83, which is identified as a *per os* infectivity infector^17^,^18^. However, as Ha83 contains only type II CBD, the *ha83* deleted HearNPV would lead to lower oral infectivity compared with wild type OBs (data not shown). This result is quite different from the function of Ac83 which would lead to no oral infectivity at all when the worms were fed with the knockout virus^17^. GP37, another typical CBD contained a protein encoded by *Spodoptera litura* multicapsid nucleopolyhedrovirus (SpltNPV) *orf32*, was reported to have chitin binding ability with a dissociation constant (K_d_) for 0.28 *μ*M^19^ which gives it an approximately two times higher efficiency to bind with chitin than Ha83 with a K_d_ value for 0.62 *μ*M (Fig. 2E). This GP37 has been shown to have pancellular dispersal in SpltNPV infected Sl-zsu-1 cells, which is very different from Ha83, for Ha83 was transported into nucleus in transfected HzAM1 cells at 48 h p.t. (Fig. 3). Interestingly, the localization of Ha83 became even in transfected cells with the sequential deletion of cysteine at the C terminal of Ha83 (Fig. 3). This is the first time to verify the CBD involved in protein subcellular localization. These results suggested that CBD encoded by *ha83* plays a different role with other reported CBD containing proteins, for Ha83 is not involved in PIF but it affects the protein transmission. And the cysteine at the 128 aa position in Ha83 CBD is probably the key conserved amino acid for CBD function.

The homologous GP37 encoded by *ac64* resembles the entomopoxvirus protein spheroidin, and has been shown to be associated with polyhedra but not with ODVs and is not essential for baculovirus replication *in vivo* or *in vitro*^20^,^21^. To investigate the functional role of *ha83* in the baculovirus life cycle, we generated a *ha83*-knockout virus. Similar to *ac64*, *ha83* was not essential for BV production (Fig. 4) but it was associated with polyhedra assembly (Fig. 5). Most of the structural gene deletion mutants, such as *ha81*, *ac93*, and *bm95* thoroughly block the BV production without disrupting viral DNA replication^10^,^22^,^23^; some reported baculovirus-host interaction genes, such as *ha132, ac68*, *sf32*, or *bm79*, do not affect the BV production and viral DNA replication simultaneously after deletion^24^,^25^,^26^,^27^; besides, some special gene knockout mutants would increase the BV production, for example, *fp25k* and *ha107*^28^,^29^. However, deletion of *ha83* resulted in an 84% decrease in BV infectivity of the bacmid virus and a 134 fold increase of DNA replicates, this peculiar change is different from any other previously reported characterized baculovirus genes, and the reasons remain unclear.

Interestingly, Ha83KO OBs were 25% larger in diameter than those of the complete virus and the shape of the occlusion bodies changed into a cuboid, whereas a 100 fold decrease in the average amount of DNA or ODVs per OB was observed in the larger occlusion bodies of *ha83* deleted HearNPV mutants. A similar diameter increase happened in *sf32* deleted SpfrMNPV, with enlargement of OBs and the nucleocapsids content reported to be 17% more in the larger *sf32* knockout SpfrMNPV OBs^26^. Other genes, such as *ac23*, modify the number of nucleocapsids per OB without affecting the number of occluded ODVs^30^,^31^. In this case, it can be assumed that the role of *ha83* in the HearNPV virus ⊠ therefore ⊠ differs from that of *ac23* in AucaMNPV and *sf32* in the SpfrMNPV. The decreased amount of ODVs in the larger Ha83KO OBs suggested that *ha83* deletion may interrupt some of the ODV associated genes expression. Not only are the OBs larger in diameter, but the morphology of Ha83KO OBs had changed into a cuboid. An abnormal morphology change of OBs was also found in the *p10* and *pep* deleted OrpsNPV mutants, as the polyhedra would become irregular in shape with pitted surfaces^32^. A *fp25k* mutant AucaMNPV isolate would assemble an abnormally large cubic polyhedron without any occluded virions inside, but this change had been verified by a single point mutation resulting in an amino acid change of Gly25 to Asp in the *polyhedrin* gene^33^. All those genes (*p10*, *pep*, and *polyhedrin*) are polyhedron associated structural genes, which directly influence the OBs morphology with any changes in expression. However, *ha83* is absolutely not an OBs structural gene, and the *ha83* deletion appears to affect the polyhedron generation in an indirect way.

Transcription qPCR analysis was performed as the *ha83* deletion affected HearNPV BV production, viral DNA replication, and OBs structure in a totally different way. Twenty ODVs and OBs structure associated genes were selected for analysis. The *polyhedrin* and *p10* transcripts were greatly reduced in Ha83KO transfected cells compared to HaWT transfected cells, which indicated that the OBs morphology change was directly caused by the low transcription level of *polyhedrin* and *p10* gene in the *ha83* knockout HearNPV. The mechanism from *ha83* that affects the *polyhedrin* and *p10* gene expression is still unknown. ODV envelope proteins are much more complex in composition than those of OBs envelopes. ODV envelope proteins are composed of Ha96 (ODV-E66)^34^, Ha73 (GP41)^35^,^36^,^37^, Ha15 (ODV-E56)^38^, Ha94 (ODV-E43)^39^, Ha44, Ha100^40^, LEF-3 (Ha65)^41^, Ha9, Helicase, Ha84^42^, VP80 (Ha92)^43^,^44^, and a group of PIFs^45^,^46^,^47^, including Ha132 (PIF2)^24^,^48^ and Ha98 (PIF3)^49^. Most of these ODV associated envelope encoding genes were decreased in transcription level with the deletion of *ha83* (Fig. 6). These results supported our hypothesis that the reduced appearance of ODVs in the enlarged *ha83* deleted OBs is partly due to the deceasing expression level of most of these ODVs associated protein encoding genes. GP41 is a tegument protein modified with O-linked *N*-acetylglucosamine located between the virion envelope and capsid^43^,^44^ that is required for the egress of nucleocapsids from the nucleus^50^. It was found to be associated with ODV by mass spectrometry^40^,^51^, and homologs are present in all baculovirus genomes. The increasing *ha73* (*gp41*) transcriptional level was contrary to the other selected genes (Fig. 6Bd). Even with more *gp41* transcripts, fewer nucleocapsids were found in the cytoplasm in *ha83* deleted bacmid transfected HzAM1 cells (Fig. 5Ab). Therefore, we doubt that the Ha83 was involved in nucleocapsids egression from nucleus, and might be somewhat related to GP41.

In conclusion, the Ha83 protein is a nonessential protein, as BV production was not blocked by gene deletion. This gene might be indirectly involved in mediating nucleocapsid organization during ODV and polyhedra assembly and occlusion. A less essential structural protein transmitted into the nucleus without the help of Ha83 might be the reason for reduced nucleocapsid production after the deletion of *ha83*.

## Methods

### Cell culture and viruses

*Helicoverpa zea* HzAM1 cells were used as host cells for viral propagation and maintained at 27 °C in TNM-FH insect medium (Sigma) supplemented with 10% fetal bovine serum (Hyclone) and 100 *μ*g/ml penicillin and 50 *μ*g/ml streptomycin. Bacmid HaBacHZ8 propagated in *Escherichia coli* strain BW25113^52^ was provided by Dr. Hu Z.H. in Wuhan Institute of Virology, CAS. Titers of BV were determined by a 50% tissue culture infective dose (TCID_50_) endpoint dilution assay in HzAM1 cells^53^.

### Total RNA preparation, RT-PCR, rapid amplification of cDNA ends (RACE) analysis

HzAM1 cells were infected by HearNPV with a multiplicity of infection (MOI) of 10 TCID_50_. Total cellular RNA was isolated using RNAiso Plus (TAKARA, JPN), according to the manufacturer’s instructions at various time points post infection (p.i.). Reverse transcription-PCR (RT-PCR) was performed with a PrimeScript^TM^ RT reagent Kit with gDNA Eraser (TAKARA) using 1.0 μg of total RNA as the template for each time point. Synthesis of first-strand DNA complementary to the mRNA (cDNA ) for the further work was carried out with oligo(dT) primers according to the manufacturer’s instructions.

The 3′ and 5′ RACE procedures were performed using 3′ and 5′-Full RACE Kits (TAKARA) with 1.0 μg of purified total RNA isolated from HearNPV infected HzAM1 cells at 48 h p.i. *Ha83* specific primers Ha835O and Ha833O (Supplementary Table S1), and nested primers, Ha835I and Ha833I (Supplementary Table S1), were used for PCR amplification. The obtained PCR products were purified and cloned into a pGEM-T easy vector (Promega, USA). Each end ten clones were selected for sequencing.

### Chitin binding assay

In order to determine the key cysteine in the CBD of Ha83, a series of different lengths of purified His-tag fused truncated Ha83 protein were generated. Six truncated Ha83 protein expression vectors (pET-28a-TC151, pET-28a-TC142, pET-28a-TC128, pET-28a-TC118, pET-28a-TC113, and pET-28a-TC71) were constructed by insertion of the truncated *ha83* PCR products into *Bam*HI and *Xho* I sites of the pET-28a(+) vector (Novagen, GER) with specific primers (Ha83-F used as forward primer, Ha83C151, Ha83C142, Ha83C128, Ha83C118, Ha83C113, and Ha83C71 were used as reverse primers, respectively) (Supplementary Table S1). pET-28a-Ha83 which can encode the entire Ha83 was constructed in a similar way with primers Ha83-F and Ha83-R. And pET-28a-ha81, which contains the entire CDs of *ha81* gene, was constructed with primers Ha81-F and Ha81-R as described above and used as the negative control plasmid for *ha81* sharing a similar base pair number with *ha83*.

The recombinant Ha83, truncated proteins (TC151, TC142, TC128, TC118, TC113, and TC71), as well as the negative control protein Ha81 were then induced by 1 mM isopropyl-β-d-thiogalactoside (IPTG) (Sigma, USA) and purified using affinity chromatography of cOmplete His-Tag Purification Resin (Roche, SUI). In the same way, all of these protein samples were investigated by 15% SDS-PAGE and stained with Coomassie Blue (Sigma). Western Blot was used for further investigation with standard protocols with the protein samples primitively separated by 10% Tricine-SDS-PAGE system^54^ and transferred to a nitrocellulose membrane. A monoclonal anti-His tag antibody (1:4000) (Abcam, GBR) was used as the primary antibodies. The horseradish peroxidase (HRP)-conjugated goat anti-mouse lgG (1:5000) (Abcam, GBR) was used as the secondary antibodies. The proteins were visualized with Enhanced HRP-DAB Chromogenic Substrate Kit (TIANGEN, CHN), according to the manufacturer’s instructions.

In order to optimize the binding conditions for the CBD proteins, the chitin powder (Sigma) was first manufactured into a chitin colloid by the following procedures: 1 g chitin powder was mixed with 40 ml HCl in a mortar and ground into a yellow homogeneous paste at 4 °C. The white chitin colloid was generated by adding the yellow mixture into 200 ml perchilled 50% ethyl alcohol and vigorously stirred for two hours. The colloid was collected by centrifugation (5000 × *g*, 5 min), and washed several times with double distilled water (ddH_2_O) until the pH value of the colloid increased to 7.1. The chitin binding assays were performed as described previously^55^,^56^. The purified protein samples were incubated with the chitin colloid at 4 °C for 16 hours. The unbounded proteins and chitin bounded proteins were separated by centrifugation (5000 × *g*, 5 min). Several wash steps were needed to reduce the unbounded proteins in the pellets. Identical volumes of the supernatant and the suspended pellet were mixed with 5 × protein loading buffer, heated for 10 min at 100 °C, and loaded onto a 10% tricine-SDS-PAGE gel. Proteins were detected by Coomassie blue staining.

In order to quantify the chitin binding activity of each protein sample, the dissociation constants detection was performed according to Kolbe *et al*.^55^,^56^. The calculated difference between the quantity of total protein initially used [T] and the unbound protein [F] allows the determination of the quantity bound to chitin [B]: [B] = [T] − [F]. In order to obtain the dissociation constants, the data were analyzed by double-reciprocal plots of [B] versus [T], as reported previously^55^,^56^. The quantity of free protein was determined by enzyme-linked immuno sorbent assay (ELISA) with monoclonal anti-His (1:4000 dilution) used as the primary antibodies and anti-(mouse IgG)-HRP used as secondary antibodies.

### Subcellular localization assays of Ha83 truncated proteins in HzAM1 cells

To determine the effect of truncated CBD on the subcellular traffic of Ha83, we generated a series of eukaryotic expression vectors in which the truncated protein was fused with EGFP. First, the 720-bp fragment containing the *egfp* gene was PCR amplified from pEGFP-N1 (Clontech, JPN) using the primers Egfp-F and Egfp-R (*Hind* III and *Xba* I sites, Supplementary Table S1). The resulting product was digested with *Hind* III III and *Xba* I, then ligated into plasmid pGL3-Basic vector which was also digested with the same mixture to generate the recombinant plasmid pGE. The *ha83* with its native promoter was PCR amplified from HaBac-HZ8 bacmid using forward primer pHa83-F and reverse primer Ha83-R and digested with *Kpn* I and *Hind* III and inserted into pGE, generating the eukaryotic expression plasmid pGE-Ha83. In a similar way, plasmids pGE-TC151, pGE-TC142, pGE-TC128, pGE-TC118, pGE-TC113, and pGE-TC71 were constructed.

1 × 10^5^ HzAM1 cells in 24-well plate (Corning life science, USA) with Thermanox^TM^ confocal coverslip (Nunc, USA) at the bottom of the well were prepared prior to transfection. 0.5 *μ*g of plasmid DNA of pGE-Ha83, pGE-TC151, pGE-TC142, pGE-TC128, pGE-TC118, pGE-TC113, and pGE-TC71 was co-transfected with 0.3 *μ*g HaBacHZ8 bacmid DNA with the X-tremeGENE HP DNA transfection reagent (Roche). At a selected time point, the coverslips were washed, fixated, and stained with 7 *μ*g/ml TRITC-Phalloidin (Sigma) and 2 *μ*g/ml 4′,6-diamidino-2-phenylindole (Dapi) (Roche). Subsequently, the cells were directly inverted on a drop of antifade mounting media (Sigma) on a glass slide and observed using a Nikon A1 confocal laser scanning microscope.

### Generation of *ha83* knockout HearNPV bacmid

The *ha83* knockout HearNPV bacmid was generated through λ-Red recombinant system reported by Hou^57^. The fragment of chloramphenicol resistant gene (*CmR*) and enhanced green fluorescent protein gene (*egfp*) were amplified with primers HaPD83F and HaPD83R (Supplementary Table S1) from PKSE vector. Homologous recombination between the 2200 bp PCR products and *ha83* was performed as previously described^10^.

Three different primers were used to confirm that *ha83* had been deleted from the *ha83* locus of the HearNPV bacmid genome. Primers Id83F and Id83R1 (Supplementary Table S1) were used to detect the correct insertion of the chloramphenicol resistant gene and *egfp*. Primers Id83F and Id83R2 (Supplementary Table S1) were used to examine the recombination junctions of the upstream and downstream flanking regions. The recombinant bacmid with all of the correct PCR confirmations was then digested by *Hind* III restriction enzyme to verify the *Hind* III-fragments changes toward the HaBacHZ8 *Hind* III digestion map. The resulting *ha83* knockout HearNPV was designated HaBac-ha83KO.

### Construction of knockout, repair, and wt HearNPV bacmids containing *polyhedron*

In order to determine if *ha83* deletion has any effect on occlusion morphogenesis, *ph* was inserted into the polyhedrin locus by site-specific transposition as previously described by Li^10^. Accordingly, the pMON7124 helper plasmid was isolated, purified, and transformed into BW25113 cells containing the HaBacHZ8 or into BW25113 cells containing the HaHZ8-ha83KO. The HTb-polh vector containing HearNPV *polyhedrin* with its own promoter^10^ was transformed into electrocompetent BW25113 cells containing the pMON7124 helper plasmid and HaHZ8-ha83KO to generate the *ha83* knockout bacmid (Ha83KO). The HTb-polh-egfp donor vector containing HearNPV *polyhedrin* and *egfp*^10^ was transformed into electrocompetent BW25113 cells containing the pMON7124 helper plasmid and HaHZ8 bacmid to generate the positive control bacmid (HaWT).

To generate a *ha83* repair bacmid, a donor plasmid (HTb-polh-ha83) was constructed. The *ha83* with its native promoter region was amplified from HearNPV with primers pha83F and ha83R (Supplementary Table S1). This fragment was introduced into the corresponding restriction sites (*Bst1107* I and *Xho* I) of the pFastBac HTb vector to generate HTb-ha83. Another fragment containing the coding region of the *ph* gene with its own promoter and the SV40 poly(A) signal was amplified with primers phS-F and phS-R (Supplementary Table S1) from the HTb-polh vector^10^. The PCR product was digested with *Bcl* I and inserted into the *Bcl* I site in HTb-ha83 to generate the final donor plasmid HTb-ha83-ph. The donor plasmid was then transformed into electrocompetent BW25113 cells harboring the pMON7124 helper plasmid and HaBac-ha83KO to generate the *ha83* repair bacmid, Ha83Rep. The correctness of transpositions were verified by PCR with primers puc/M13F and puc/M13R (Supplementary Table S1), and followed by PCR product sequencing.

### One step viral growth curve and viral DNA replication analysis

To analyze BV production, a viral growth curve was constructed as previously described^10^. Titers of BV were determined by a TCID_50_ assay and compared by F-test in SPSS 15.0 (SPSS Inc, Chicago, IL). To evaluate any effect of *ha83* deletion on viral DNA replication, qPCR analysis was performed as previously described^10^.

### Electron microscopy

HzAM1 cells (10^6^ cells per 35-mm-diameter well) were transfected with 1.0 *μ*g of the constructed bacmid HaWT, Ha83KO, or Ha83Rep. At 72 h p.i., cells were dislodged with a rubber policeman and centrifuged at 2000 × g for 10 min. Occlusion bodies HaWT and Ha83KO were washed and resuspended in 100 μl of 2% agarose. The OBs/agarose pellets were washed in double sterile water and sliced into 2 mm cubes. Both the cells and OBs were fixed in 2.5% glutaraldehyde over night, and then dehydrated, embedded, sectioned, and stained as described previously^10^. Samples were examined with a Hitachi HT7700 transmission electron microscope at an accelerating voltage of 80 kV. For analysis by scanning electron microscopy, occlusion bodies were coated with gold and examined with a Hitachi 5X-300000X scanning electron microscope. OB measurements were normalized by square root transformation and compared by t-test using SPSS 15.0.

### Transcriptional analysis of HearNPV occlusion body and ODV associated genes

To further examine the effects of *ha83* knockout, transcripts from several well-characterized polyhedron and ODV associated structural genes were analyzed. HzAM1 cells (106 cells per 35-mm-diameter well) were transfected with 1.0 *μ*g of the constructed bacmid DNA ha83KO and HaWT as described above. Total RNA extraction and cDNA synthesis from transfected cells at 24, 48, and 72 h p.t. was performed as described above. As target genes, we selected *polyhedrin* and *p10* as representative OBs structural genes. *pif2, ha9, an, ha100, odv-e66, p24, cg30, odv-e56,lef-3, vp80, ha66, ha40, helicase, odv-e43, ha90, ie-1, 38k*, and *gp41* were selected as representative ODV associated structural genes. qPCR was then performed as described above, using the primers listed in Supplementary Table S1. The relative expression level was compared by F-test using SPSS 15.0.

## Uncited reference

## Additional Information

**How to cite this article**: Yu, H. *et al*. *Ha83*, a Chitin Binding Domain Encoding Gene, Is Important to *Helicoverpa armigera* Nucleopolyhedrovirus Budded Virus Production and Occlusion Body Assembling. *Sci. Rep*. **5**, 11088; doi: 10.1038/srep11088 (2015).

## Supplementary Material

Supplementary Information

## References

[b1] Herniou E. A., Olszewski J. A., Cory J. S. & O’Reilly D. R. The genome sequence and evolution of baculoviruses. Annu. Rev. Entomol. 48, 211–234 (2003). 12414741 10.1146/annurev.ento.48.091801.112756

[b2] Braunagel S. C. & Summers M. D. *Autographa californica* nuclear polyhedrosis virus, PDV, and ECV viral envelopes and nucleocapsids: structural proteins, antigens, lipid and fatty acid profiles. Virology 202, 315–328 (1994). 8009843 10.1006/viro.1994.1348

[b3] Slack J. & Arif B. M. The baculoviruses occlusion-derived virus: virion structure and function. Adv. Virus Res. 69, 99–165 (2007). 17222693 10.1016/S0065-3527(06)69003-9 PMC7112300

[b4] Van Oers M. M. & Vlak J. M. Baculovirus genomics. Curr. Drug Targets. 8, 1051–1068 (2007). 17979665 10.2174/138945007782151333

[b5] Slack J. M., Dougherty E. M. & Lawrence S. D. A study of the *Autographa californica* multiple nucleopolyhedrovirus ODV envelope protein p74 using a GFP tag. J. Gen. Virol. 82, 2279–2287 (2001). 11514740 10.1099/0022-1317-82-9-2279

[b6] Chen X. W. . The sequence of the *Helicoverpa armigera* single nucleocapsid nucleopolyhedrovirus genome. J. Gen. Virol. 82, 241–257 (2001). 11125177 10.1099/0022-1317-82-1-241

[b7] Chen X. W., Hu Z. H., Jehle J. A. & Vlak J. M. Analysis of the Ecdysteroid UDP-Glucosyltransferase Gene of *Heliothis armigera* Single-Nucleocapsid Baculovirus. Virus Genes. 15, 219–225 (1997). 9482587 10.1023/a:1007976420021

[b8] Chen X. W. . Identification, sequence analysis and phylogeny of the lef-2 gene of *Helicoverpa armigera* single-nucleocapsid baculovirus. Virus Res. 65, 21–32 (1999). 10564750 10.1016/s0168-1702(99)00097-0

[b9] Wang H. L. . Characterization and phylogenetic analysis of the chitinase gene from the *Helicoverpa armigera* single nucleocapsid nucleopolyhedrovirus. Virus Res. 100, 179–189 (2004). 15019236 10.1016/j.virusres.2003.11.015

[b10] Li X. F., Yu H., Zhang C. X., Chen H. & Wang D. *Helicoverpa armigera* nucleopolyhedrovirus *orf81* is a late gene involved in budded virus production. Arch. Virol. 159, 2011–2022 (2014). 24623087 10.1007/s00705-014-2034-2

[b11] Dong C. S. . Identification of functional domains required for HearNPV P10 filament formation. Virology 338, 112–120 (2005). 15936792 10.1016/j.virol.2005.05.003

[b12] Long G., Westenberg M., Wang H. L., Vlak J. M. & Hu Z. H. Function, oligomerization and N-linked glycosylation of the *Helicoverpa armigera* single nucleopolyhedrovirus envelope fusion protein. J. Gen. Virol. 87, 839–846 (2006). 16528033 10.1099/vir.0.81592-0

[b13] Wang D. & Zhang C. X. HearSNPV *orf* 83 encodes a late nonstructural protein with an active chitin-binding domain. Virus Res. 117, 237–243 (2006). 16313991 10.1016/j.virusres.2005.10.019

[b14] Tao X. Y. . The *Autographa californica* Multiple nucleopolyhedrovirus ORF78 is essential for budded virus production and general occlusion body formation. J. Virol. 87, 8441–8450 (2013). 23698311 10.1128/JVI.01290-13 PMC3719795

[b15] Todd J. W., Passarelli A. L., Lu A. & Miller L. K. Factors regulating baculovirus late and very late gene expression in transient-expression assays. J. Virol. 70, 2307–2317 (1996). 8642657 10.1128/jvi.70.4.2307-2317.1996 PMC190072

[b16] Tellam R. L., Wijffels G. & Willadsen P. Peritrophic matrix proteins. Insect Biochem. Mol. Biol. 29, 87–101 (1999). 10.1016/s0965-1748(98)00123-4 10196732

[b17] Zhu S. M., Wang W., Wang Y., Yuan M. J. & Yang K. The baculovirus core gene *ac83* is required for nucleocapsid assembly and per of infectivity of *Autographa californica* Nucleopolyhedrovirus. J. Virol. 87, 10573–10586 (2013). 23864639 10.1128/JVI.01207-13 PMC3807414

[b18] Peng K. . Characterization of novel components of the baculovirus *per os* infectivity factor complex. J. Virol. 86, 4981–4988 (2012). 22379094 10.1128/JVI.06801-11 PMC3347349

[b19] Li Z. F. . Characterization of a chitin-binding protein GP37 of *Spodoptera litura* multicapsid nucleopolyhedrovirus. Virus Res. 96, 113–122 (2003). 12951271 10.1016/s0168-1702(03)00179-5

[b20] Vialard J. E., Yuen L. & Richardson C. D. Identification and characterization of a baculovirus occlusion body glycoprotein which resembles spheroidin, an entomopoxvirus protein. J. Virol. 64, 5804–5811 (1990). 2243377 10.1128/jvi.64.12.5804-5811.1990 PMC248735

[b21] Cheng X. W., Krell P. J. & Arif B. M. P34.8 (GP37) is not essential for baculovirus replication. J. Gen. Virol. 82, 299–305 (2001). 11161266 10.1099/0022-1317-82-2-299

[b22] Yuan M. J. . Identification of *Autographa californica* nucleopolyhedrovirus *ac93* as a core gene and its requirement for intranuclear microvesicle formation and nuclear egress of nucleocapsids. J. Virol. 85, 11664–11674 (2011). 21880748 10.1128/JVI.05275-11 PMC3209287

[b23] Xiang X. W. . *Bombyx mori* nucleopolyhedrovirus *BmP95* plays an essential role in budded virus production and nucleocapsid assembly. J. Gen. Virol. 94, 1669–1679 (2013). 23535571 10.1099/vir.0.050583-0

[b24] Fang M. G. . Open reading frame 132 of *Heliocoerpa armigera* nucleopolyhedrovirus encodes a functional *per os* infectivity factor (PIF-2). J. Gen. Virol. 87, 2563–2569 (2006). 16894194 10.1099/vir.0.81788-0

[b25] Dong Z. Q. . *Bombyx mori* nucleopolyhedrovirus ORF79 is a *per os* infectivity factor associated with the PIF complex. Virus Res. 184, 62–72 (2014). 24583368 10.1016/j.virusres.2014.02.009

[b26] Beperet I. . The *sf32* unique gene of *Spodoptera frugiperda* multiple nucleopolyhdrovirus (SfMNPV) is a non-essential gene that could be involved in nucleocapsid organization in occlusion-derived virions. Plos One 8, e77683 (2013). 24204916 10.1371/journal.pone.0077683 PMC3813766

[b27] Nie Y. C., Fang M. G., Erlandson M. A. & Theilmann D. A. Analysis of the *Autographa californica* multiple nucleopolyhedrovirus overlapping gene pair *lef3* and *ac68* reveals that AC68 is a *per os* infectivity factor and that LEF3 is critical, but not essential, for virus replication. J. Virol. 83, 3985–3994 (2012). 22278232 10.1128/JVI.06849-11 PMC3302525

[b28] Wu D. . Functional analysis of FP25K of *Helicoverpa armigera* single nucleocapsid nucleopolyhdrovirus. J. Gen. Virol. 86, 2439–2444 (2005). 16099901 10.1099/vir.0.81110-0

[b29] Pan X. Y. . Deletion of a *Helicoverpa armigera* nucleopolyhedrovirus gene encoding a virion structural protein (ORF107) increases the budded virion titre and reduces *in vivo* infectivity. J. Gen. Virol. 88, 3307–3316 (2007). 18024900 10.1099/vir.0.83363-0

[b30] Yu L. L., Bray D., Lin Y. C. & Lung O. *Autogtrapha californica* multiple nucleopolyhedrovirus ORF 23 null mutant produceds occlusion-derived virions with fewer nucleocapsids. J. Gen. Virol. 90, 1499–1504 (2009). 19264654 10.1099/vir.0.009035-0

[b31] Lung O. Y. . Ac23, an envelope fusion protein homolog in the baculovirus *Autographa californica* multicapsid nucleopolyhderovirus, is a viral pathogenicity factor. J. Virol. 77, 328–339 (2003). 12477838 10.1128/JVI.77.1.328-339.2003 PMC140606

[b32] Gross C. H., Russell R. L. & Rohrmann G. F. *Orgyia pseudotsugata* baculovirus p10 and polyhedron envelope protein genes: analysis of their relative expression levels and role in polyhedron structure. J. Gen. Virol. 75, 1115–1123 (1994). 8176372 10.1099/0022-1317-75-5-1115

[b33] Lin G. Y., Zhong J. & Wang X. Z. Abnormal formation of polyhedra resulting from a single mutation in the polyhedrin gene of *Autographa californica* multicapsid nucleopolyhedrovirus. J. Invertebr. Pathol. 76, 13–19 (2000). 10963398 10.1006/jipa.2000.4934

[b34] Hong T., Braunagel S. C. & Summers M. D. Transcription, translation, and cellular localization of PDV-E66: a structural protein of the PDV envelope of *Autographa californica* nuclear polyhedrosis virus. Virology 201, 201–222 (1994). 10.1006/viro.1994.1525 8091653

[b35] Whitford M. & Faulkner P. A structural polypeptide of the baculovirus *Autographa californica* nuclear polyhedrosis virus contains O-linked *N*-acetylglucosamine. J. Virol. 66, 3324–3329 (1992). 1583718 10.1128/jvi.66.6.3324-3329.1992 PMC241110

[b36] Whitford M. & Faulkner P. Nucleotide sequence and transcriptional analysis of a gene encoding gp41, a structural glycoprotein of the baculovirus *Autographa californica* nuclear polyhedrosis virus. J. Virol. 66, 4763–4768 (1992). 1629955 10.1128/jvi.66.8.4763-4768.1992 PMC241303

[b37] Liu J. C. & Maruniak J. E. Molecular characterization of genes in the GP41 region of baculoviruses and phylogenetic analysis based upon GP41 and polyhedrin genes. Virus Res. 64, 187–196 (1999). 10518714 10.1016/s0168-1702(99)00094-5

[b38] Braunagel S. C., Elton D. M., Ma H. & Summers M. D. Identification and analysis of an *Autographa californica* nuclear polyhedrosis virus structural protein of the occlusion-derived virus envelope: ODV-E56. Virology 317, 97–110 (1996). 8599240 10.1006/viro.1996.0097

[b39] Fang M. G. . Open reading frame 94 of *Helicoverpa armigera* single nucleocapsid nucleopolyhedrovirus encodes a novel occlusion-derived virion protein, ODV-EC43. J. Gen. Virol. 84, 3021–3027 (2003). 14573807 10.1099/vir.0.19291-0

[b40] Deng F. . Poteomics analysis of *Helicoverpa armigera* single nucleocapsid nucleopolyhedrovirus identified two new occlusion-derived virus-associated proteins, Ha44 and Ha100. J. Virol. 81, 9377–9385 (2007). 17581982 10.1128/JVI.00632-07 PMC1951453

[b41] Kool M., Ahrens C. H., Goldbach R. W., Rohrmann G. F. & Vlak J. M. Identification of genes involved in DNA replication of the *Autographa californica* baculovirus. P. Natl. Acad. Sci. USA. 91, 11212–11216 (1994). 10.1073/pnas.91.23.11212 PMC45197 7972036

[b42] Chen X. W. . Identification, sequence analysis and phylogeny of the lef-2 gene of *Helicoverpa armigera* single-nucleocapsid baculovirus. Virus Res. 65, 21–32 (1999). 10564750 10.1016/s0168-1702(99)00097-0

[b43] Lu A. & Carstens E. B. Nucleotide sequence and transcriptional analysis of the p80 gene of *Autographa californica* nuclear polyhedrosis virus: a homologue of the *Orgyia pseudotsugata* nuclear polyhedrosis virus capsid-associated gene. Virology 190, 201–209 (1992). 1529529 10.1016/0042-6822(92)91206-a

[b44] Muller R., Pearson M. N., Russell R. L. O. & Rohrmann G. F. A capsid-associated protein of the multicapsid nuclear polyhedrosis virus of *Orgyia pseudotsugata*: genetic location, sequence, transcriptional mapping, and immunocytochemical characterization. Virology 176, 133–144 (1990). 2184573 10.1016/0042-6822(90)90238-m

[b45] Faulkner P., Kuzio J., Williams G. V. & Wilson J. A. Analysis of p74, a PDV envelope protein of *Autographa californica* nucleopolyhedrovirus required for occlusion body infectivity *in vivo*. J. Gen. Virol. 78, 3091–3100 (1997). 9400957 10.1099/0022-1317-78-12-3091

[b46] Kuzio J., Jaques R. & Faulkner P. Identification of p74, a gene essential for virulence of baculovirus occlusion bodies. Virology 173, 759–763 (1989). 2688302 10.1016/0042-6822(89)90593-x

[b47] Yao L. G., Zhou W. K., Xu H., Zheng Y. & Qi Y. P. The *Heliothis armigera* single nucleocapsid nucleopolyhedrovirus envelope protein P74 is required for infection of the host midgut. Virus Res. 104, 111–121 (2004). 15246648 10.1016/j.virusres.2004.03.005

[b48] Pijlman G. P., Pruijssers A. J. P. & Vlak J. M. Identification of pif-2, a third conserved baculovirus gene required for per os infection of insects. J. Gen. Virol. 84, 2041–2049 (2003). 12867634 10.1099/vir.0.19133-0

[b49] Ohkawa T., Washburn J. O., Sitapara R., Sid E. & Volkman L. E. Specific Binding of *Autographa californica M* Nucleopolyhedrovirus Occlusion-Derived Virus to Midgut Cells of *Heliothis virescens* Larvae Is Mediated by Products of *pif* Genes *Ac119* and *Ac022* but Not by *Ac115*. J. Virol. 79, 15258–15264 (2005). 16306597 10.1128/JVI.79.24.15258-15264.2005 PMC1316039

[b50] Olszewski J. & Miller L. K. A role for baculovirus GP41 in budded virus production. Virology 233, 292–301 (1997). 9217053 10.1006/viro.1997.8612

[b51] Braunagel S. C., Russell W. K., Rosas-Acosta G., Russell D. H. & Summers M. D. Determination of the protein composition of the occlusion-derived virus of *Autographa californica* nucleopolyhedrovirus. P. Natl. Acad. Sci. USA. 100, 9797–802 (2003). 10.1073/pnas.1733972100 PMC187845 12904572

[b52] Wang H. Z. . Cloning of biologically active genomes from a *Helicoverpa armigera* single-nucleocapsid nucleopolyhedrovirus isolate by using a bacterial artificial chromosome. Virus Res. 97, 57–63 (2003). 14602197 10.1016/j.virusres.2003.07.001

[b53] King L. A. & Possee R. D. The baculovirus expression system: a laboratory guide. Cambridge University Press, London (1992).

[b54] Hermann S. Tricine-SDS-PAGE. Nat. Protoc. 1, 16–22 (2006). 17406207 10.1038/nprot.2006.4

[b55] Kolbe S., Fischer S., Becirevic A., Hinz P. & Schrempf H. The *Streptomyces reticuli* α-chitin-binding protein CHB2 and its gene. Microbiology 144, 1291–1297 (1998). 9611804 10.1099/00221287-144-5-1291

[b56] Zeltins A. & Schrempf H. Specific interaction of the *Streptomyces* chitin-binding protein CHB1 with α-chitin. The role of individual tryptophan residues. Eur. J. Biochem. 246, 557–564 (1997). 9208950 10.1111/j.1432-1033.1997.t01-1-00557.x

[b57] Hou S., Chen X., Wang H., Tao M. & Hu Z. Efficient method to generate homologous recombinant baculovirus genomes in *E. coli*. BioTechniques 32, 783–788 (2002). 11962600 10.2144/02324st04

