# Supplementary material for: Ha83, a Chitin Binding Domain Encoding Gene, Is Important to Helicoverpa armigera Nucleopolyhedrovirus Budded Virus Production and Occlusion Body Assembling
Source: Sci Rep. 2015 Jun 9;5:11088. doi: 10.1038/srep11088 (PMC4460901; doi:10.1038/srep11088)
Supplement: Supplementary Information [file srep11088-s1.pdf]

SUPPLEMENTAL INFORMATION

Supplementary Figure 1-5

Supplementary Table 1

Ha83, a Chitin Binding Domain Encoding Gene, Is Important to *Helicoverpa armigera* Nucleopolyhedrovirus Budded Virus Production and Occlusion Body Assembling

Huan Yu[a,b], Jian Xu[b], Qiang Liu[a], Tong-Xian Liu[a,b]*, Dun Wang[a,b]*

a. State Key Laboratory of Crop Stress Biology for Arid Areas Northwest A&F University, Yangling, Shaanxi, P. R. China

b. Key Laboratory of Applied Entomology, Northwest A&F University, Yangling, Shaanxi, P. R. China

* Corresponding author.

* Correspondence to Tong-Xian Liu, txliu@nwsuaf.edu.cn

* Correspondence to Dun Wang, wanghande@yahoo.com; wanghande@nwsuaf.edu.cn;

Address: Institute of Entomology, Northwest A&F University, Yangling, Shaanxi 712100, P.R. China.

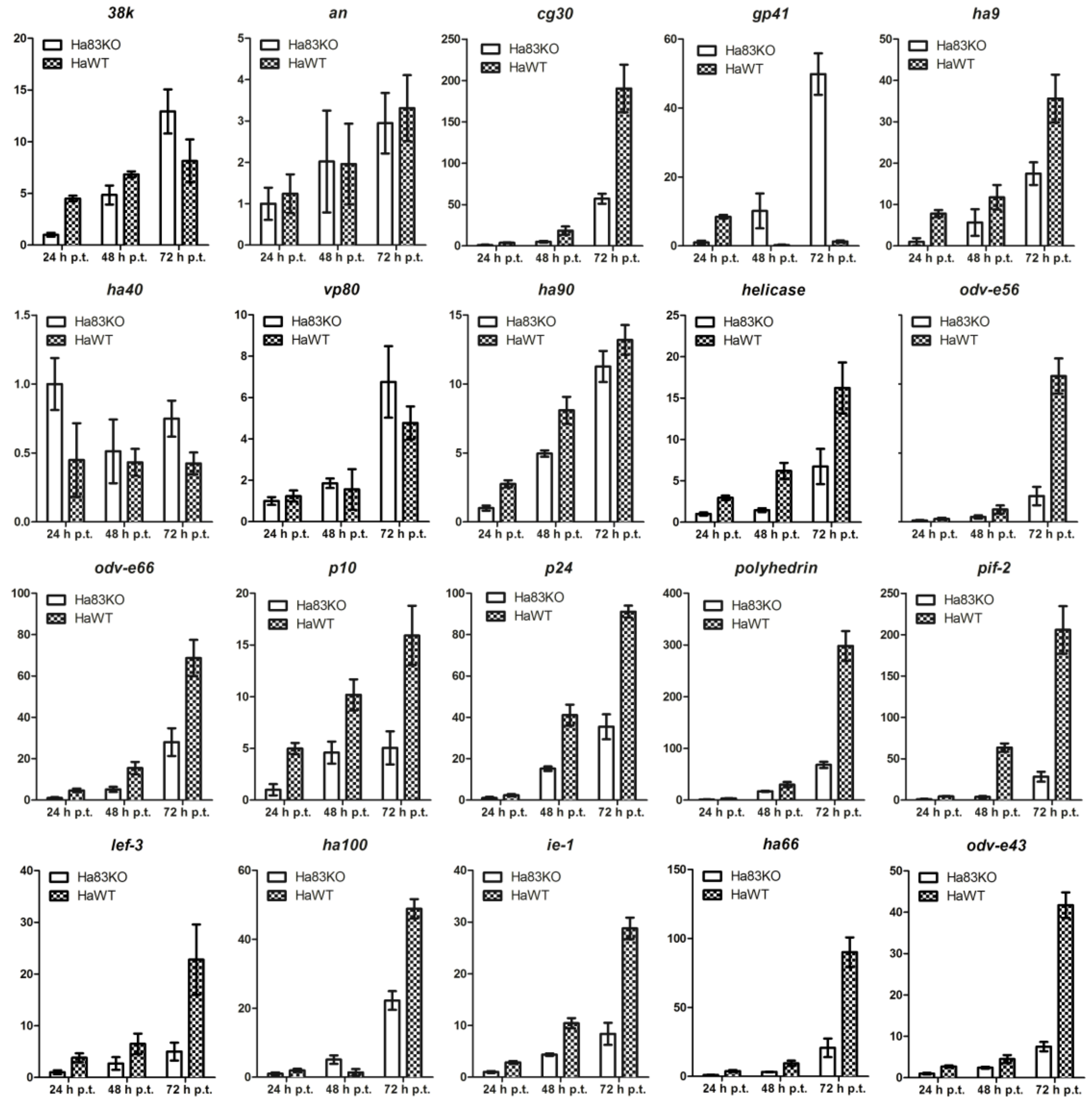

Supplementary Figure S1. Relative expression of viral genes in Ha83KO or HaWT transfected HzAM1 cells. Error bars represent the standard deviation.

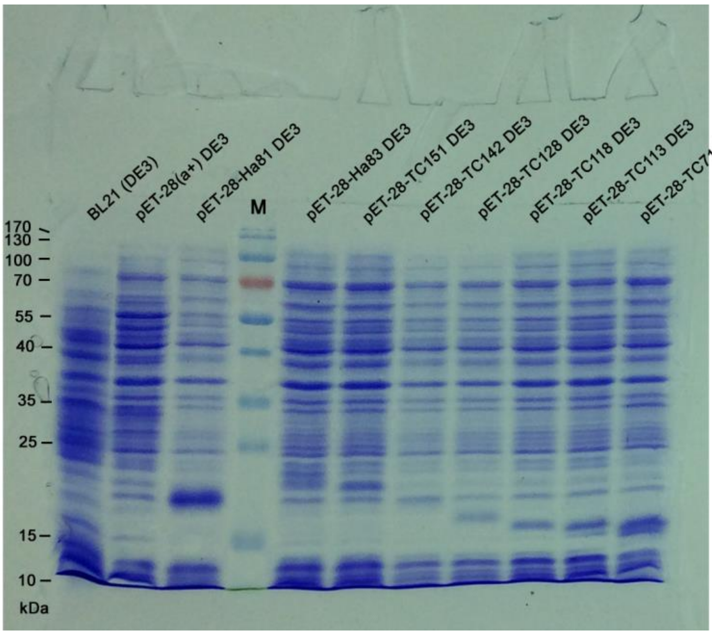

Supplementary Figure S2. Uncropped gel version for Figure 2A in the main text. The protein samples of Ha83 and truncated mutants expressed by *E. coli* BL21(DE3) was loaded on a 15% SDS-PAGE gel, and the gel was stained with Coomassie blue.

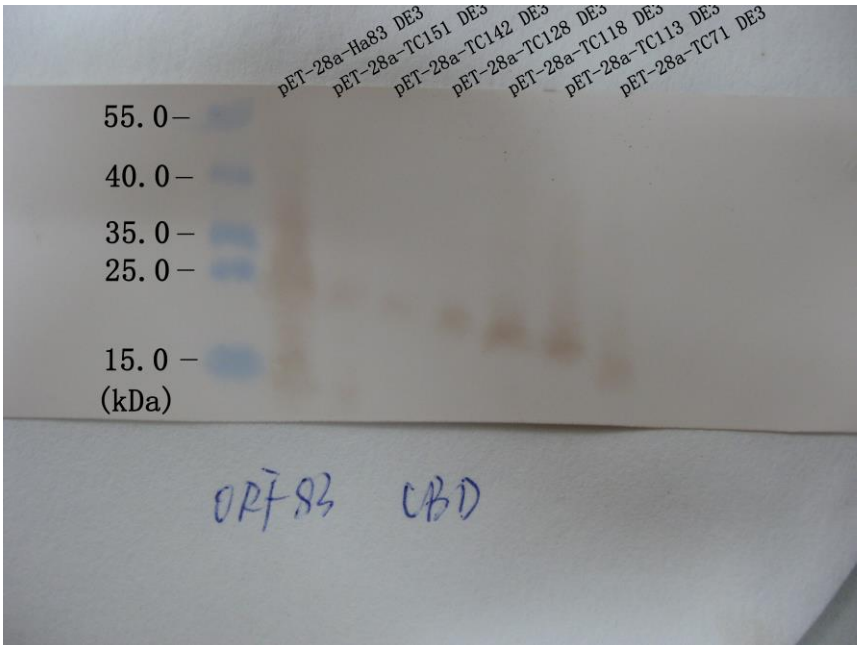

Supplementary Figure S3. Uncropped Blot version for Figure 2B in the main text. The protein samples of Ha83 and truncated mutants expressed by *E. coli* BL21(DE3) was loaded on a 10% Tricine SDS-PAGE gel, and followed by a standard Western bolt

protocols.

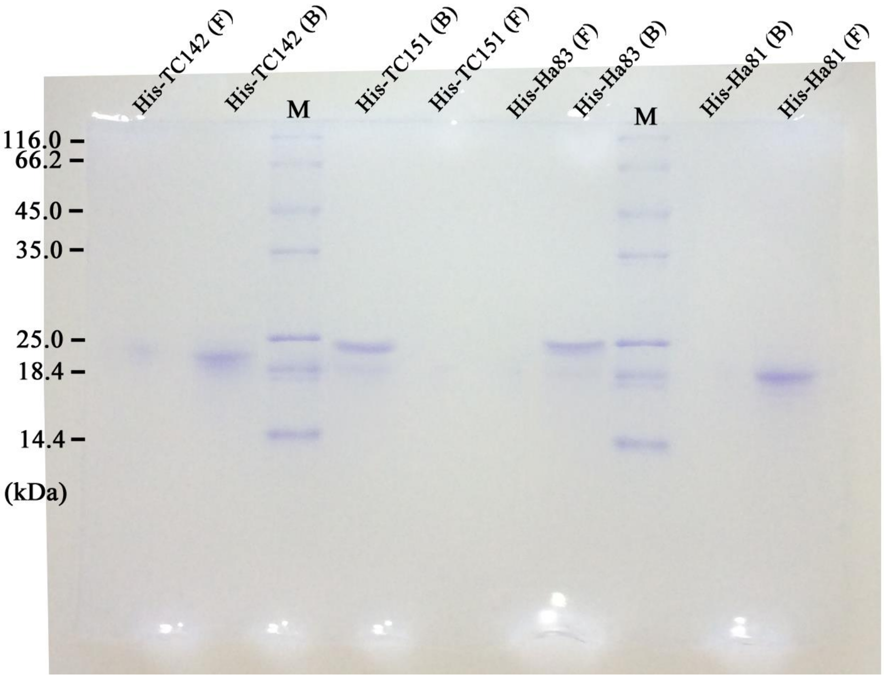

Supplementary Figure S4. Uncropped gel version for Figure 2C in the main text.. The bound protein (B, bound protein) and unbound protein in the supernatant (F, free protein) of Chitin binding tests on His-Ha83, His-Ha81, His-TC151, His-TC142 were loaded in each lane of a 10% Tricine-SDS-PAGE system, respectively.

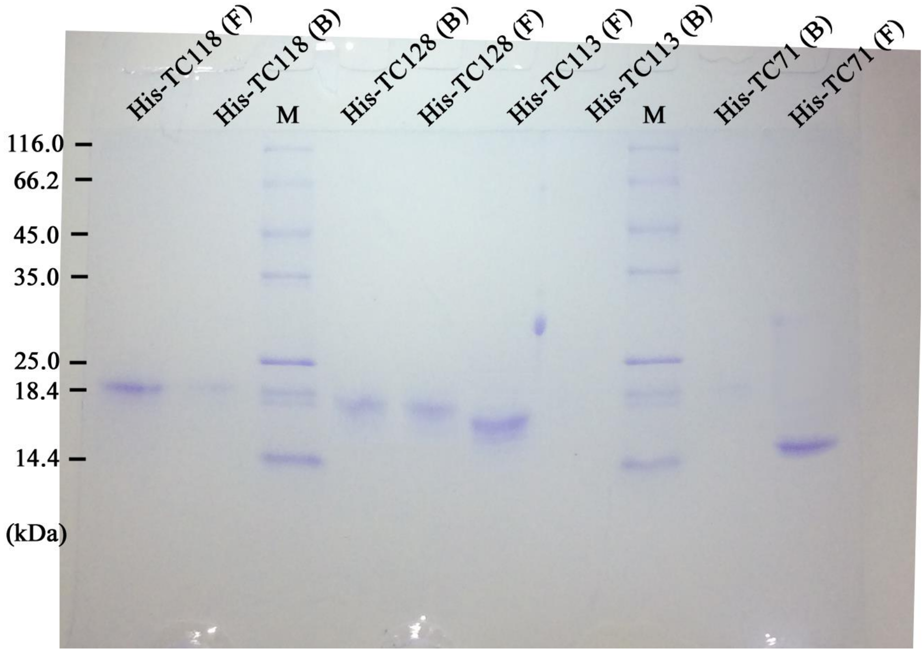

Supplementary Figure S5. Uncropped gel version for Figure 2C in the main text. The bound protein (B, bound protein) and unbound protein in the supernatant (F, free protein) of Chitin binding tests on His-TC128, His-TC118, His-TC113, His-TC71 were loaded in each lane of a 10% Tricine-SDS-PAGE system, respectively.

Supplementary Table S1. Primers used in this study

Primer	Sequence[a]	Position (nt)	Amplification purpose
Ha835I	5’- GTCATCATCGGGCGGTGTTACTGGT -3’	75890→75914	Inner primer for *ha83* 5’ RACE analysis.
Ha835O	5’- ACGTGCAACGTTTTTTCAGGGC -3’	75801→75822	Outer primer for *ha83* 5’ RACE analysis.
Ha833I	5’- TACAGCAATGATGTTGCGATGTTCTGAT -3’	75641←75668	Inner primer for *ha83* 3’ RACE analysis.
Ha833O	5’- ACAATGCGATAATTACTGGGGC -3’	75719←75740	Outer primer for *ha83* 3’ RACE analysis.
Ha83-F	5’- G GGATCC ATGAGTCCTCAATATGCAATA -3’ (*BamH* Ⅰ)	75993←76013	Forward primer of complete *ha83* from HearNPV for prokaryotic expression vector construction.
pha83F	5’- T AAGCTT GTATAC AAATCTTAAAATCGGATTCG -3’ (*Kpn* Ⅰ /*Bst1107* Ⅰ)	76387←76402	Forward primer of *ha83* and its promoter from HearNPV for eukaryotic expression vector construction.
Ha83-R	5’- G CTCGAG TTA AAGCTT	75534→75551	Reverse primer of complete *ha83* from

	TTTTATAAATTTTTTTATT -3’ (*Xho* Ⅰ / *Hind*Ⅲ)		HearNPV for pET-28a-Ha83 construction.
Ha83C151	5’- G CTCGAG TTA AAGCTT TTTGTTATCGTTTAATGT -3’ (*Xho* Ⅰ / *Hind*Ⅲ)	75582→75599	Reverse primer of the first 450 bp of *ha83* from HearNPV for truncation vector construction.
Ha83C142	5’- G CTCGAG TTA AAGCTT ATCGTCTATTTCTGGATC-3’ (*Xho* Ⅰ / *Hind*Ⅲ)	75609→75626	Reverse primer of the first 423 bp of *ha83* from HearNPV for truncation vector construction.
Ha83C128	5’- G CTCGAG TTA AAGCTT TCGCAACATCATTGCTGT-3’ (*Xho* Ⅰ / *Hind*Ⅲ)	75648→75665	Reverse primer of the first 384 bp of *ha83* from HearNPV for truncation vector construction.
Ha83C118	5’- G CTCGAG TTA AAGCTT CAAGATAA ACTTTTGAC-3’ (*Xho* Ⅰ / *Hind*Ⅲ)	75678→75694	Reverse primer of the first 354 bp of *ha83* from HearNPV for truncation vector construction.

Ha83C113	5’- G CTCGAG TTA AAGCTT ATCATATTTATGCGGTAA-3’ (*Xho* Ⅰ / *Hind*Ⅲ)	75696→75713	Reverse primer of the first 336 bp of *ha83* from HearNPV for truncation vector construction.
Ha83C71	5’- G CTCGAG TTA AAGCTT TTGTACGGTACAATCGCACGA -3’ (*Xho* Ⅰ / *Hind* Ⅲ)	75735→75755	Reverse primer of the first 297 bp of *ha83* from HearNPV for truncation vector construction.
Egfp-F	5’- G AAGCTT ATGGTGAGCAAGGGCGAGGAGCT -3’ (*Hind* Ⅲ)		Amplification of complete *egfp* region from pEGFP-N1 vector.
Egfp-R	5’- G TCTAGA TTACTTGTACAGCTCGTCCATG -3’ (*Xba* Ⅰ)		
Ha81-F	5’- CGGATCCATGACCTCGTCACAGGAGCAA -3’ (*BamH* Ⅰ)		Amplification of complete *ha81* from HearNPV for prokaryotic expression vector

	5’- GCTCGAGCTCAAACAATTTGTATGACA -3’		construction as a negative control.
Ha81-R	(*Xho* I)		
	5’-		Amplification of *Cm*^{r} and *egfp* with HearNPV
	GTATACCTAATTGTGGCCATGTTGTCTCATTTTAT		*ha83* flanking sequences.
	GTCCAA	75944←75997	
	ACCTATAATACCA		
HaPD83F	TAACTCGAGAAATTTCTCTGGCCG -3’		
	5’-		
	TGTATTGACACAATCGTCTATTTCTGGATCGAAAC		
	AAAAG	75599→75652	
	GAATCAGAACATCG		
HaPD83R	AAGCTTTTTAACGGCACCAATA -3’		
Id83F	5’- TGTGCAATTGAAACTCAAGG -3’	75411→75430	Identification of *ha83* knockout bacmid.

Id83R1	5’- GCCCGATGATGACGACAATC -3’	75883←75902	
Id83R2	5’- GAGTCCTCAATATGCAATAC -3’	76012←76031	
phS-F	5’ – TGATCA AATATGAAGATTTCTGTCGTCGTGTTG – 3’ (*Bcl* Ⅰ)		Amplification of *polyhedrin* with its promoter and SV40 polyA signal from HTb-polh.
phS-R	5’ – TGATCA GATCCAGACATGATAAGATACATTGATG – 3’ (*Bcl* Ⅰ)		
ph-F	5’- CGGATCC ATGTATACTCGTTACAGT -3’ (*BamH* Ⅰ)	1→18	Amplification of complete *polyhedrin* from HearNPV.
ph-R	5’- ACTGCAG TTAATATGCAGGACCAGT -3’ (*Pst* Ⅰ)	724←741	
puc/M13-F	5’- CCCAGTCACGACGTTGTAAAACG -3’		Identification of transposition.

puc/M13-R	5’- AGCGGATAACAATTTCACACAGG -3’		
qGp41-F	5’- CATCCGATTAGCGTGAACG -3’	66316←66334	*gp41* specific primer for qPCR.
qGp41-R	5’- GGGCATAACTCGGCAACAC -3’	66181→66199	
qPH-F	5’- GTTACAGTTACAGCCCTACTTTGG -3’	11→34	*polyhedrin* specific primer for qPCR.
qPH-R	5’- CTTCTTGCGTTTGGCATTTT -3’	89←108	
qP10-F	5’- TGTCGGTGCTGGTTGATAAGAT -3’	18463←18484	*p10* specific primer for qPCR.
qP10-R	5’- TCATCGGCAGGAGGTATCGTA -3’	18373→18393	
qIE-1 F	5’- GAGTCGGATAGTAGCAGTTTGG -3’	11837→11858	*ie-1* specific primer for qPCR.
qIE-1 R	5’- ACTTCGTTGAGTGGCGTTGC -3’	11945←11964	
qHa44 F	5’- GCAATTTATCGGCGGGATTT -3’	39454→39473	*ha44* specific primer for qPCR.
qHa44 R	5’- CAAACGGCGACGACAAGTAA -3’	39582←39601	
qLEF-3 F	5’- TTACGGAACGCAAACTCAAT -3’	56190→56209	*lef-3* specific primer for qPCR.
qLEF-3 R	5’- GCATACGGTCATCAACAGGTC -3’	56305←56325	

qHa66 F	5’- AAAATATCTTCAGTAGCGTCCAAG -3’	59398→59421	*ha66* specific primer for qPCR.
qHa66 R	5’- TAAATTCAGGCACGGTCCAG -3’	59466←59485	
qCG30 F	5’- TGTTCGGACATTTAACCTCT -3’	70823→70842	*cg30* specific primer for qPCR.
qCG30 R	5’- ATTGTCAAGTGCCCTCTGTG -3’	70930←70949	
qP24 F	5’- TCAGAGTCAAATTGTCGATCCGTTGT -3’	113075→113100	*p24* specific primer for qPCR.
qP24 R	5’- GCTGGGCTGTGGTCGGCATA -3’	113183←113202	
qODV-E43 F	5’- GATTACAGCGACATAAACAACAGC -3’	88271→88294	*odv-e43* specific primer for qPCR.
qODV-E43 R	5’- GTAACGCATTGCCGACACTC -3’	88353←88372	
qODV-E56 F	5’- TAATACGACACCTACTCCTACTAGACCCAC -3’	13948→13977	*odv-e56* specific primer for qPCR.
qODV-E56 R	5’- CAGCCGAAGGCGTCACCAAT -3’	14038←14057	
qHa90 F	5’- CGGCGTCGGATCTTTTAGTA -3’	83878→83897	*ha90* specific primer for qPCR.
qHa90 R	5’- ATGTCAGCGAAGTGCCCATT -3’	83947←83966	
qVP80 F	5’- TCAACCGTCGCCGTTCACTT -3’	86190→86209	*vp80* specific primer for qPCR.

qVP80 R	5'- TTGCGTTTGCGTCGCACTTT -3'	86296←86315	
qHa100 F	5'- GACGTGACGGTAGCCCTGTA -3'	95767→95786	*ha100* specific primer for qPCR.
qHa100 R	5'- AGACGGTGTCGCTGCCTAAT -3'	95862←95881	
qODV-E66 F	5'- GTCCCACCACCGAACCAAAG -3'	89740→89759	*odv-e66* specific primer for qPCR.
qODV-E66 R	5'- AGCACGACAGGGTAGCAACTA -3'	89850←89870	
qAN F	5'- TGACCAACGACGAATCCAGT -3'	107366→107385	*an* specific primer for qPCR.
qAN R	5'- ACGCTCGCAAAGAACGCTAC -3'	107490←107509	
qHelicase F	5'- CCCATCGTTCGTTGAAACTC -3'	78352→78371	*helicase* specific primer for qPCR.
qHelicase R	5'- AAATCCTTGCGTGCTACTCG -3'	78427←78446	
qHa9 F	5'- TGTCTCATACAGGGCAATACG -3'	8276→8296	*ha9* specific primer for qPCR.
qHa9 R	5'- AATACAAACGATAGGGAAAGG -3'	8379←8399	
qPIF-2 F	5'- TGTGCCGTTACATTCGTTGC -3'	126788→126807	*pif-2* specific primer for qPCR.
qPIF-2 R	5'- ATTGGGCGTTTCCACTGGTC -3'	126886←126905	

[a] Homologous parts were italicized, restriction enzyme sites were underlined.
